# TiO*_x_*/Pt_3_Ti(111) surface-directed formation of electronically responsive supramolecular assemblies of tungsten oxide clusters

**DOI:** 10.3762/bjnano.12.16

**Published:** 2021-02-16

**Authors:** Marco Moors, Yun An, Agnieszka Kuc, Kirill Yu Monakhov

**Affiliations:** 1 Peter Grünberg Institute, Department of Electronic Materials, Forschungszentrum Jülich GmbH, Wilhelm-Johnen-Str., 52425 Jülich, Germany; 2 Leibniz Institute of Surface Engineering (IOM), Permoserstraße 15, 04318 Leipzig, Germany; 3 Helmholtz-Zentrum Dresden-Rossendorf, Department of Reactive Transport, Institute of Resource Ecology, Permoserstraße 15, 04318 Leipzig, Germany

**Keywords:** atom manipulation, scanning tunneling microscopy, supramolecular self-assemblies, titanium oxide, tungsten oxide

## Abstract

Highly ordered titanium oxide films grown on a Pt_3_Ti(111) alloy surface were utilized for the controlled immobilization and tip-induced electric field-triggered electronic manipulation of nanoscopic W_3_O_9_ clusters. Depending on the operating conditions, two different stable oxide phases, z’-TiO*_x_* and w’-TiO*_x_*, were produced. These phases show a strong effect on the adsorption characteristics and reactivity of W_3_O_9_ clusters, which are formed as a result of thermal evaporation of WO_3_ powder on the complex TiO*_x_*/Pt_3_Ti(111) surfaces under ultra-high vacuum conditions. The physisorbed tritungsten nano-oxides were found as isolated single units located on the metallic attraction points or as supramolecular self-assemblies with a W_3_O_9_-capped hexagonal scaffold of W_3_O_9_ units. By applying scanning tunneling microscopy to the W_3_O_9_–(W_3_O_9_)_6_ structures, individual units underwent a tip-induced reduction to W_3_O_8_. At elevated temperatures, agglomeration and growth of large WO_3_ islands, which thickness is strongly limited to a maximum of two unit cells, were observed. The findings boost progress toward template-directed nucleation, growth, networking, and charge state manipulation of functional molecular nanostructures on surfaces using operando techniques.

## Introduction

Molecular electronics has developed to a fast-growing research field in the past decades. Aspects such as low-cost fabrication and potentially high scalability down to the level of single molecules, resulting directly in a very low power consumption, make this type of electronic devices appealing for the implementation in conceptually new data storage cells [1]. Specifically, resistive memories [2] based on the change of the electrical properties of a transition metal oxide thin layer, integrated in a simple electrode setup as a function of an externally applied potential [3], have shown great potential for next-generation information technologies. This change of the electrical resistance often faces local redox reactions inside the oxide layer [4]. From the chemical point of view, the active switching layer can be downsized to individual molecular units (e.g., polyoxometalates [5–6]) with many distinct and stable redox states. Recently, representatives of this interesting chemical class have also been shown to act as stable single-molecule three‐state transistors [7].

However, controlled adsorption and molecular ordering on surfaces remain significant points of focus for their integration into practical devices. In fact, the economically ideal way to obtain molecule-based devices would be the propensity of surface-deposited molecular metal-oxide clusters to self-assemble toward stable and reproducible functional supramolecular structures [8]. One possibility to achieve this is to create substrate surfaces, whose characteristics would give rise to the nanostructured metal-oxide self-assembly formation.

Herein, we demonstrate the potential of this approach using thermal evaporation of WO_3_ powder under ultra-high vacuum (UHV) on ultrathin titanium oxide layers grown on a Pt_3_Ti(111) surface. The given case study with the nanoscopic W_3_O_9_ clusters as the main components of tungsten trioxide vapor, which have been evidenced by mass spectrometric studies [9–10], showcases remarkable TiO*_x_*/Pt_3_Ti(111)-directed reactivity of W_3_O_9_ to hierarchical supramolecular structures. It is noteworthy that individual W_3_O_9_ clusters, according to previous density functional theory (DFT) calculations [11], are characterized by the most stable six-membered ring structure with *D*_3_*_h_* symmetry. It consists of oxygen-bridged tungsten atoms with two additional terminal oxygen atoms per each W^VI^ center. As a result, tungsten ions are in tetrahedral coordination environments. W_3_O_9_ shows a large energy gap of 3.4 eV which nearly reaches the value of bulk WO_3_ exhibiting a direct band gap of 3.5 eV [12]. Overall, W_3_O_9_ can be seen as the smallest molecular model for bulk WO_3_. However, the resulting formation of W_3_O_9_ by thermal WO_3_ evaporation under UHV conditions differs significantly from other WO_3_ deposition techniques. For example, the formation of hydrated tungsten acid species could be demonstrated by electrochemical evaporation of tungsten oxide on rutile surfaces under aqueous conditions [13].

Theoretical studies were not only performed for neutral W_3_O_9_ clusters but also for their oxygen-deficient and anionic derivatives. Hereby, an energetic stabilization caused by a significant d-orbital aromaticity was found for [W_3_O_9_]^−^ and [W_3_O_9_]^2−^ [14], which is an indication of an experimental evidence of these species by external charge injection. Theoretically interesting are also under-coordinated W_3_O_9_ derivatives, such as W_3_O_8_ with *C_S_* symmetry, in which one of the tungsten centers has only one terminal oxygen atom instead of two. From simulations it is known that such a tri-coordinated tungsten site possesses a localized 5d electron pair and, thus, can be regarded as an oxygen-deficient defect site [11]. This results in a significantly increased oxygen adsorption energy of −78 kcal·mol^−1^ for the W_3_O_8_ cluster.

During the past decades, W_3_O_9_ clusters were investigated on different surfaces by using high-resolution scanning tunneling microscopy (STM), in particular on TiO_2_(110) [15–16], CuO(110) [17], and Pt(111) [18]. Recently, the surface behavior of W_3_O_9_ was assessed on a complex CuWO_3_ phase grown on Cu(110). The CuWO_3_/Cu(110) substrate can be viewed as a two-dimensional (2D) ternary oxide layer [19], which is based on a mixed Cu–O top layer with an alignment of Cu atoms along the [100] direction [20]. The Cu surface rows were shown to act as preferential adsorption sites for W_3_O_9_ nanoclusters. In all the above-mentioned studies, the W_3_O_9_ clusters were imaged with submolecular resolution as a triangular structure representing the unoccupied electronic states of the tungsten atoms. On pure oxide surfaces the clusters showed no substantial long-range order and they tended to form agglomerates at higher coverages [15–17]. In contrast to that, the W_3_O_9_ clusters on Pt(111) formed nearly ordered ultrathin films due to the stronger interfacial interaction; however, only in the second layer. In the first layer, an opening of the ring structure resulted in a zigzag chain structure [18]. These findings, thus, showcase the strong effect of a solid support on the surface behavior of W_3_O_9_.

To stabilize and utilize different charge states of the cluster outside the gas phase, we have prepared a substrate material, which, on the one hand, offers a sufficient template effect for the self-organization of the nanoclusters and, on the other hand, exhibits a rather low electronic surface interaction. Both characteristics are implemented by atomically ordered titanium oxide ultrathin films grown on a bimetallic Pt_3_Ti(111) single crystal surface. More importantly, the use of this material has already proven successful in supporting the self-organization of Pd clusters [21].

Exposing the Pt_3_Ti alloy surface to different oxygen doses at elevated temperatures results in the formation of several atomically thin and highly ordered TiO*_x_* phases, which were described in detail elsewhere [22–23]. Depending on the preparation conditions, up to four different TiO*_x_* phases can be prepared on the Pt_3_Ti(111) surface. For this work, only two thermodynamically stable oxide phases were utilized: (i) the z’-TiO*_x_* phase with a rectangular unit cell, which is characterized by a typical stripe pattern originated from titanium atoms with different oxygen coordination, and (ii) the w’-TiO*_x_* phase with a wagon-wheel-like structural motif. The complex pattern of this hexagonally shaped titanium oxide film is due to the moiré effect on the underlying metallic substrate. Both phases consist of an oxygen-terminated bilayer with a stoichiometric TiO phase [22], such that the structural differences are triggered by different surface coverages. While the rectangular z’-TiO*_x_* phase is a typical island structure, whose number and size increase with the oxygen dosage, the hexagonal w’-TiO*_x_* phase always covers the entire surface. In the latter case, the surface stress would be too high to engage the usually favored rectangular symmetry of titanium oxides. Therefore, the TiO layer is forced to adopt the hexagonal symmetry of the substrate.

Note that the formation of the oxide z’- and w’-TiO*_x_* phases is not only limited to the growth on the Pt_3_Ti(111) surface shown herein. Sedona et al. [24–26] demonstrated, in many points, identical oxide phases by electron beam evaporation of Ti on Pt(111) at increased oxygen partial pressures. However, the direct oxidation of the Pt_3_Ti alloy surface has the advantage of an easier reproducibility and an increased long-range order of the individual oxide phases [22].

For this study, theory-supported STM measurements at liquid nitrogen temperatures have been selected as an ideal characterization technique. This is due to the fact that it not only allows high-resolution imaging on the nanoscale, but the STM tip may also act as a charge-injecting or depleting electrode for the controlled manipulation of single W_3_O_9_ clusters. The power of the STM approach for molecular switches has been demonstrated several times. For example, Cui et al. recently showed the reversible switching of a large discoid polyaromatic salt as a function of the applied bias voltage [27]. By that, we were able to show the outstanding interaction of W_3_O_9_ and different oxide phases formed on the Pt_3_Ti(111) surface.

## Results and Discussion

### W_3_O_9_ adsorption on the z’-TiO*_x_* phase grown on Pt_3_Ti(111)

**Figure 1:**
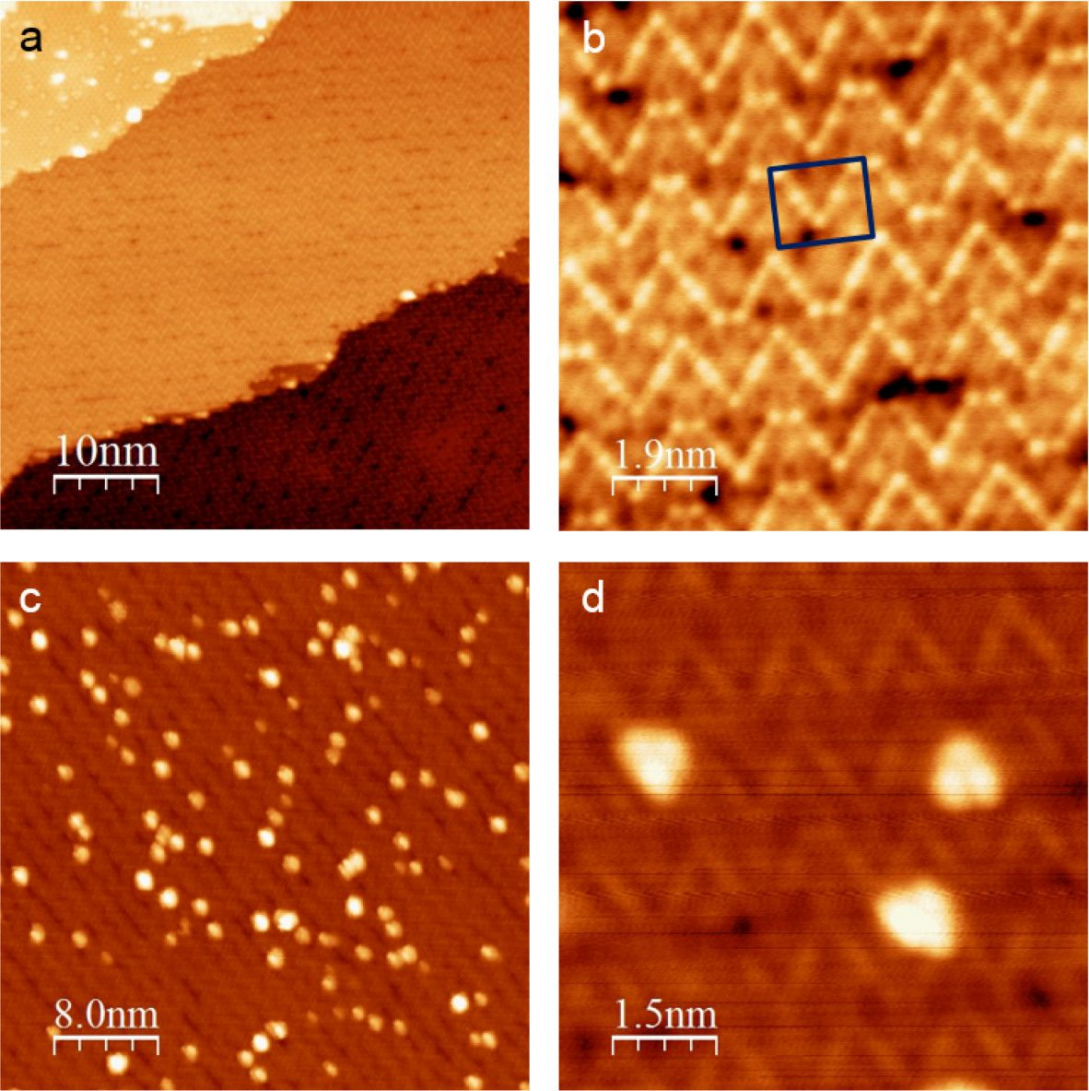
STM images of the z’-TiO*_x_* phase grown on Pt_3_Ti(111): (a) An overview image (50 × 50 nm; *U*_B_ = 1.57 V; *I*_T_ = 160 pA) of the clean oxide phase. In the upper left area, oxide-free patches of the metallic substrate can still be found. (b) An atomic resolution scan (9.4 × 9.4 nm; *U*_B_ = 1.22 V; *I*_T_ = 160 pA) of the z’-TiO*_x_* phase with a marked unit cell. The dark areas inside the trenches represent holes in the oxide film. (c) The z’-TiO*_x_* phase after deposition of W_3_O_9_ (≈0.1 monolayer coverage; 40 × 40 nm; *U*_B_ = 1.00 V; *I*_T_ = 86 pA). The clusters are always aligned along the trenches. (d) A submolecular resolution scan (7.7 × 7.7 nm; *U*_B_ = 1.00 V; *I*_T_ = 86 pA) of individual W_3_O_9_ clusters.

Figure 1a and Figure 1b show atomically resolved STM images of the z’-TiO*_x_* phase. Under the employed preparation conditions, most terraces are completely covered with the zigzag structure of the oxide. Only few oxide-free patches can be found, which show the characteristic titanium atom-related hexagonal structure of the alloy substrate with a lattice vector of 5.5 ± 0.1 Å [28] (see upper left corner of Figure 1a). From LEED and STM measurements, a commensurate rectangular unit cell with a (6×3√3) superstructure with respect to the (1×1) spots of the alloy surface and with a size of 16.6 ± 0.2 Å × 14.4 ± 0.2 Å was determined [22–23]. The zigzag lines are approximately 1.4 nm apart from each other and separated by darker trenches. As shown in Figure 1b, the stripes consist of an irregular sequence of v- and w-shaped structural motifs. Hereby, the side of a v motif is always build up by four bright atoms with diverging interatomic distances, while the side of a w motif consists of only three bright atoms with a constant distance between them. The DFT calculations of a very similar oxide phase, grown by a reactive Ti deposition on Pt(111) [25], showed that the brightest spots belong to fourfold oxygen-coordinated titanium atoms, whereas threefold coordinated Ti atoms are depicted with less contrast. The combination of these two species forms the characteristic stripe pattern of the z’-TiO*_x_* phase. Inside the trenches between the stripes, several holes in the film can be found, which are probably caused by the stress release during the film growth. These defects permit a direct contact with the underlying substrate and, thus, they should be special attraction points for adsorbates.

As shown in Figure 1c and Figure 1d, it is very probable that W_3_O_9_ clusters prefer to adsorb these defects inside the trenches of the z’-TiO*_x_* phase. The result is a 1D alignment of the clusters along the direction of the stripes. Hereby, the clusters appear in the typical triangular form with a side length of 13.0 ± 0.5 Å and a maximum height of 2.0 ± 0.2 Å. An STM tip-induced manipulation of single clusters was not possible within practically manageable voltage and current limits. The rather high stability may be due to a strong interaction with the substrate. The exclusive positioning of the W_3_O_9_ clusters on the point defects inside the trenches leads us to the conclusion that the preferred interaction with the metallic substrate below the oxide film compensates any charge injection or removal induced by the STM tip. For that purpose, an oxide surface with a lower defect concentration should be more promising.

### W_3_O_9_ adsorption on the w’-TiO*_x_* phase grown on Pt_3_Ti(111)

**Figure 2:**
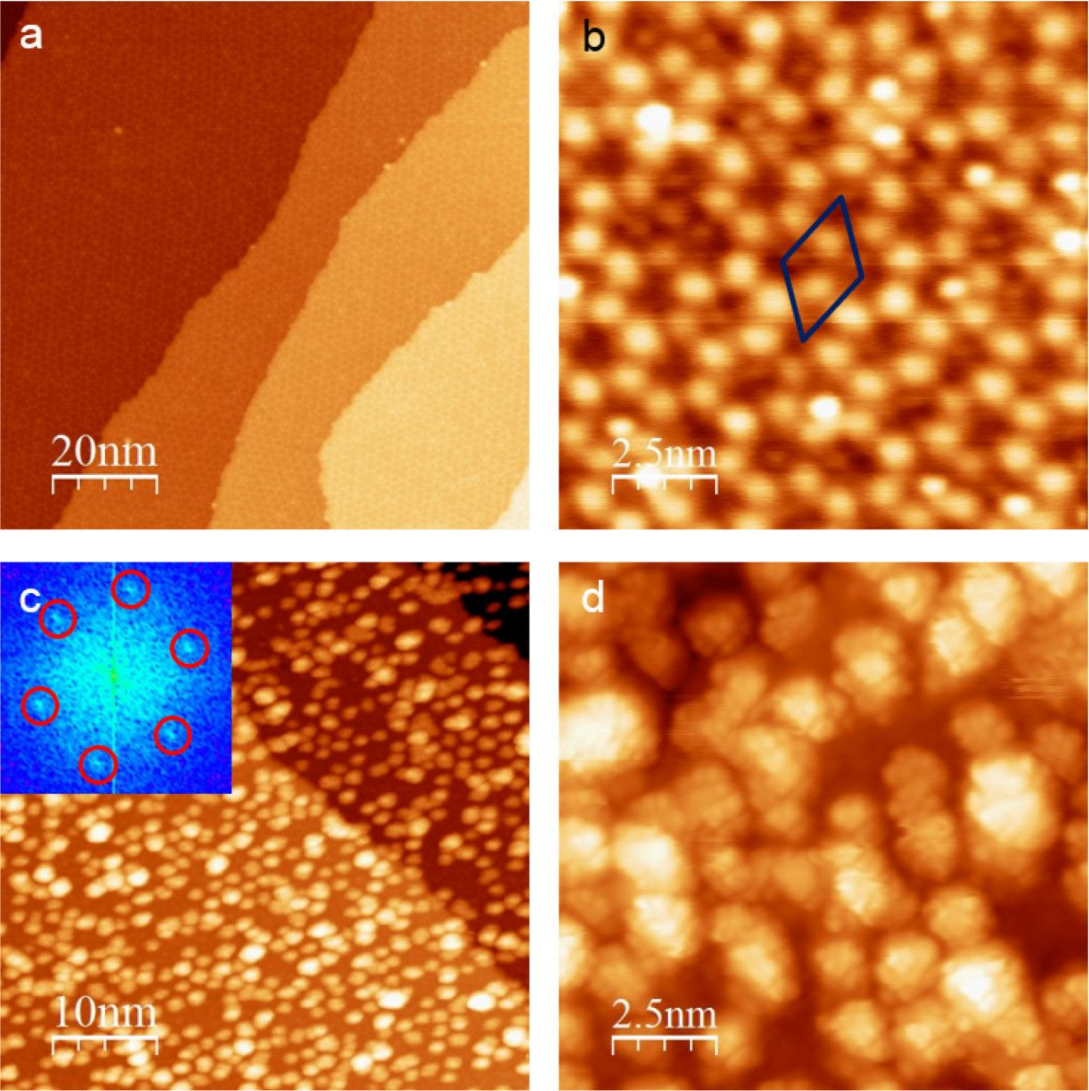
STM images of the w’-TiO*_x_* phase grown on Pt_3_Ti(111): (a) An overview image (100 × 100 nm; *U*_B_ = 2.50 V; *I*_T_ = 62 pA) of the clean oxide phase. The oxide film covers the entire surface. (b) A high-resolution scan (50 × 50 nm; *U*_B_ = 1.57 V; *I*_T_ = 100 pA) of the w’-TiO*_x_* phase with a marked unit cell. The bright spots represent the coincidence points in the moiré structure caused by the interplay between the oxide layer and the substrate. (c) The w’-TiO*_x_* phase after deposition of W_3_O_9_ (≈0.6 monolayer coverage; 50 × 50 nm; *U*_B_ = 1.57 V; *I*_T_ = 58 pA). The clusters show a hexagonal arrangement (see marked spots in the FFT inset) with a slight tendency to form agglomerations. (d) A submolecular resolution scan (7.7 × 7.7 nm; *U*_B_ = 1.00 V; *I*_T_ = 62 pA) of individual W_3_O_9_ clusters.

Figure 2a and Figure 2b show the less defective w’-TiO*_x_* phase with its characteristic wagon-wheel structure caused by a moiré pattern of the Ti–O bilayer and the metallic substrate. As expected, oxide-free areas can no longer be found on the surface. Former LEED measurements indicated a commensurate hexagonal (7×7)R21.8° superstructure with a lattice vector of 19.4 ± 0.2 Å [22], which was also verified using STM [23]. By superimposing a hexagonal TiO layer with a unit cell size of 3.18 Å, which is rotated by 3.5° on the alloy substrate with an interatomic Pt atom distance of 2.76 Å, the observed moiré pattern can be simulated [23,26]. In contrast to the z’-TiO*_x_* phase, the amount of surface holes is negligibly low, although the oxide film is still atomically thin. Thus, a direct contact of the adsorbates with the metallic alloy surface can be excluded.

As shown in Figure 2c and Figure 2d, the adsorption of W_3_O_9_ on the hexagonal w’-TiO*_x_* phase shows a behavior that is completely different from the behavior observed on the rectangular z’-phase. On the fully closed oxide film, a hexagonal arrangement of larger particles can be detected (see the FFT inset in Figure 2c). Hereby, the hexagonal coincidence points of the oxide moiré pattern seem to act as preferred nucleation centers. Different from the adsorption on the z’-TiO*_x_* phase, there is still a slight tendency to form agglomerations indicating a weaker overall interaction with the w’-TiO*_x_* phase. This is quite intuitive because, in the latter case, the clusters have no direct contact with the metallic substrate due to the completely closed oxide film.

**Figure 3:**
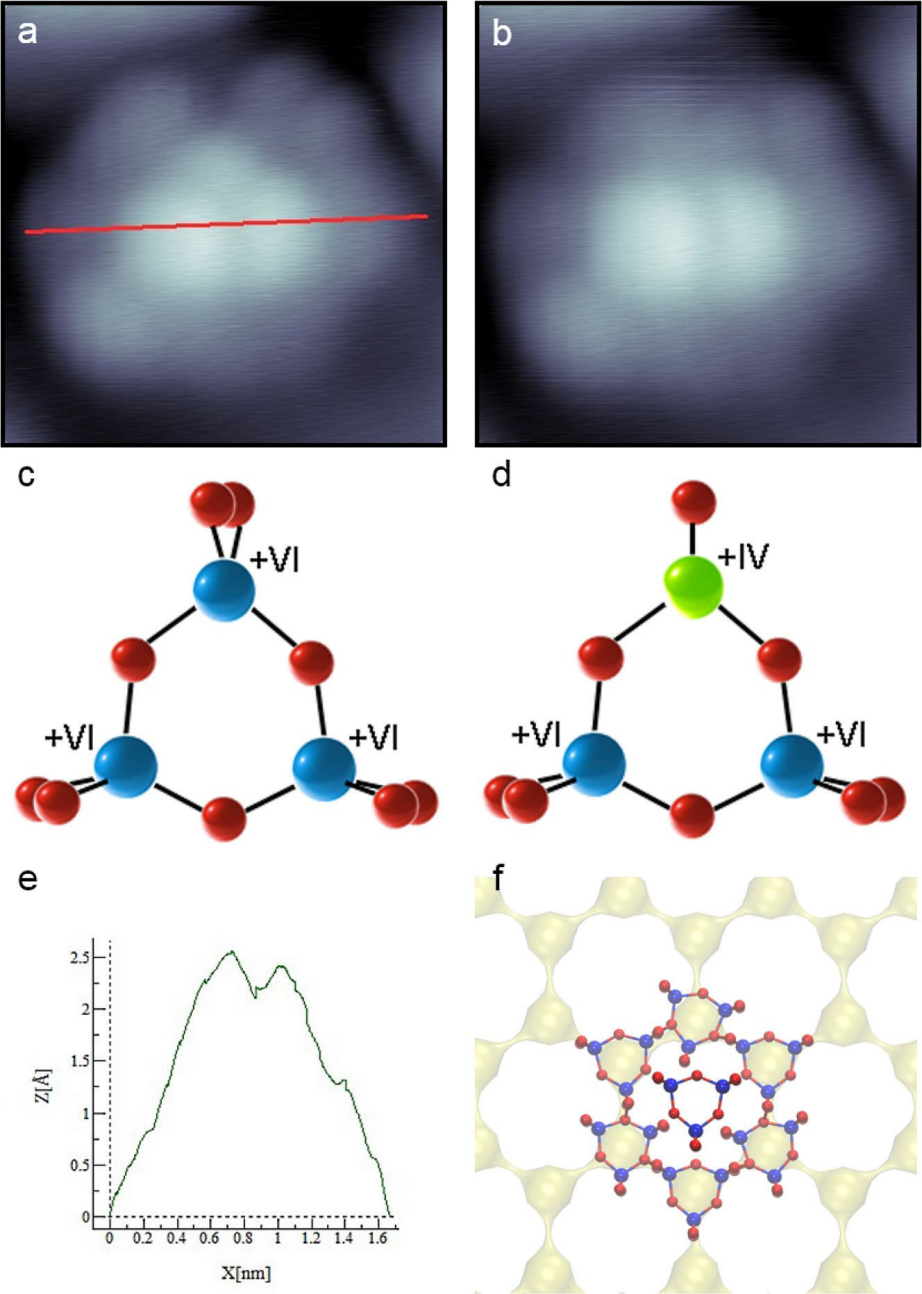
High-resolution STM images (both 1.8 × 1.8 nm; *U*_B_ = 0.50 V; *I*_T_ = 62 pA) of a supramolecular assembly of W_3_O_9_ molecules on the w’-TiO*_x_* phase grown on Pt_3_Ti(111) before (a) and after (b) an intermediate scan with *U*_B_ = −1.00 V and *I*_T_ = 40 pA. By this procedure, the appearance from the top-laying molecule changed from a three-lobe to a two-lobe structure, indicating a partial reduction of one of the tungsten atoms resulting in W_3_O_8_. Panels (c) and (d) illustrate the proposed structures before and after the procedure. The graph in (e) shows the corresponding height profile of the W_3_O_9_ stack in (a) marked by the red line. Panel (f) illustrates the proposed orientation on the w’-TiO*_x_* phase grown on Pt_3_Ti(111).

**Figure 4:**
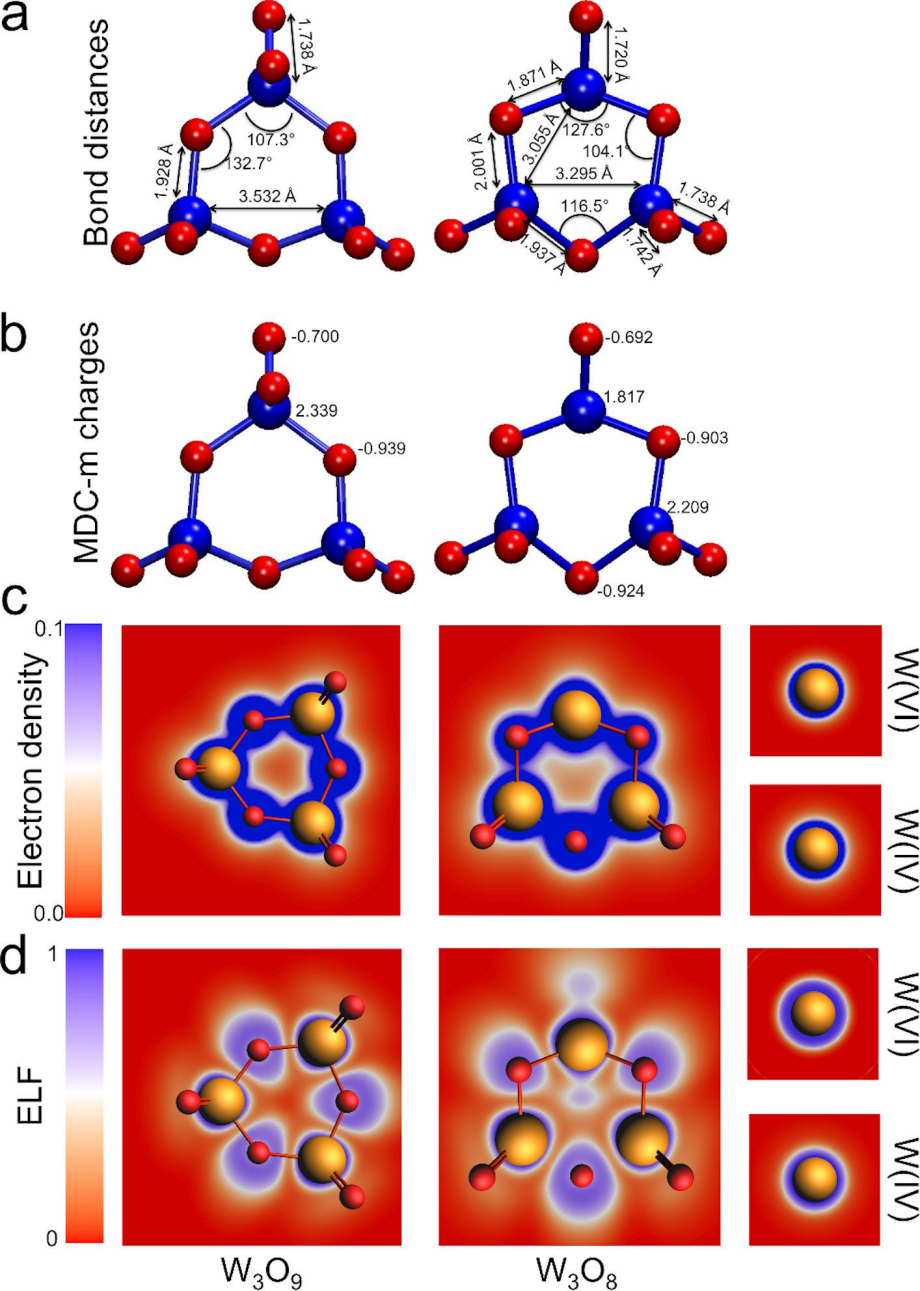
Structural and electronic properties of simulated W_3_O_9_ and W_3_O_8_ clusters. (a) Relaxed structures with the respective *D*_3_*_h_* and *C_s_* symmetries and the selected bond distances and bond angles. (b) Multipole-derived charges using monopoles (MDC-m) of all atoms, showing that two types of W atoms are present in the W_3_O_8_ cluster, corresponding to +VI and +IV oxidation states. (c) Total electron density (ρ) and (d) electron localization function (ELF). The latter two show differences in the electron density and localization not only for W atoms but also for O atoms within the hexagonal ring.

High-resolution STM images (Figure 3a) reveal very complex particles with a hexagonal symmetry and a brighter triangle of electron density depicted in the center, which is characteristic of W_3_O_9_. Since form and size of the outer lobes of these particles are rather similar to the brighter ones in the center, we proposed a supramolecular assembly of single W_3_O_9_ clusters as the most probable explanation. Hereby, six clusters shape again a hexagonal framework, on top of which a seventh W_3_O_9_ is adsorbed (Figure 3f). The rather exact doubled height of the inner lobes (Figure 3e) indicates a physical rise of the inner cluster, although electronic effects cannot be fully excluded. By considering simple geometrical aspects, one supports the idea that in a closed packed hexagonal arrangement of W_3_O_9_ there is not enough space left for an additional cluster in the center without overlapping. In order to exclude possible tip–surface deconvolution effects (e.g., by a random W_3_O_9_ pickup) it should be noted that neither soft pulsing procedures of the STM tip during the measurements nor a complete tip exchange had a significant influence on the observations. However, it is well known that the coincidence points of the w’-TiO*_x_* phase on the Pt_3_Ti alloy substrates exhibit unique electronic properties [21,23]. Thus, it is not surprising that the adsorption on such positions may result in extraordinary arrangements. As a consequence of this supramolecular assembly, the risen cluster in the center does not have a direct contact with the metallic substrate or with the titanium oxide layer due to the formed W_3_O_9_ interlayer. In consequence, this only indirect bonding to the surface leads to an electronic state, which should be very near to that of an isolated molecule in the gas phase. Indeed, our experiments with the STM tip exhibited a behavior that had never been observed for W_3_O_9_ immobilized on a surface. Figure 3b shows a single W_3_O_9_ cluster after an intermediate image scan with a negative bias voltage of −1.0 V, resulting in an effective current flow from the negatively charged sample to the positively charged tip. Obviously, this treatment has a significant effect on the structural appearance of the cluster assembly. The bright cluster depicted on top of the assembly changed its appearance from a threefold to a twofold symmetry, indicating a loss of electron density in one of the tungsten centers. This structural change in the electron density distribution can be explained by a partial reduction of the respective tungsten atom by the tip-induced charge flow from the surface. In consequence, one of the outer W=O double bonds should be weakened or even completely broken. In the latter case, this would result in a formal W_3_O_8_ cluster, in which two tungsten atoms remain in the oxidation state +VI and one is reduced to +IV (compare Figure 3c and Figure 3d). The latter one has less unoccupied electronic states and, therefore, is not resolved by scanning with a positive bias voltage. Our DFT calculations of W_3_O_9_ and of a hypothetical W_3_O_8_ molecule in the gas phase strongly support this theory (see Figure 4). The calculated geometries of both clusters are *D*_3_*_h_* and *C_s_* for W_3_O_9_ and W_3_O_8_, respectively. The bond length distribution strongly changes, especially close to W^IV^. This results in strongly changed electronic properties. Two different oxidation states are obtained from the atomic charge analysis, corresponding to +IV and +VI in W_3_O_8_. Electron density and electron localization function show a slight increase for the W^IV^ atom compared to the W_3_O_9_ cluster.

In addition, the orientation of one of the W_3_O_9_ clusters in the layer below has obviously changed (compare the upper right areas of Figure 3a and Figure 3b). We assume that this effect is caused by the unsealed oxygen ion, which is absorbed by the oxygen-depleted TiO*_x_* layer and, thus, locally disturbs the order of the above-lying W_3_O_9_ layer. However, the expected high adsorption energy of oxygen in W_3_O_8_ should also lead to an increased local interaction with the transition metal oxide layer, which might play a role in the new assembly arrangement. We have observed stronger interaction of both clusters with a simpler TiO_2_ surface, in which O atoms from the cluster interact with Ti atoms of the surface, while W atoms interact with O atoms of the surface.

**Figure 5:**
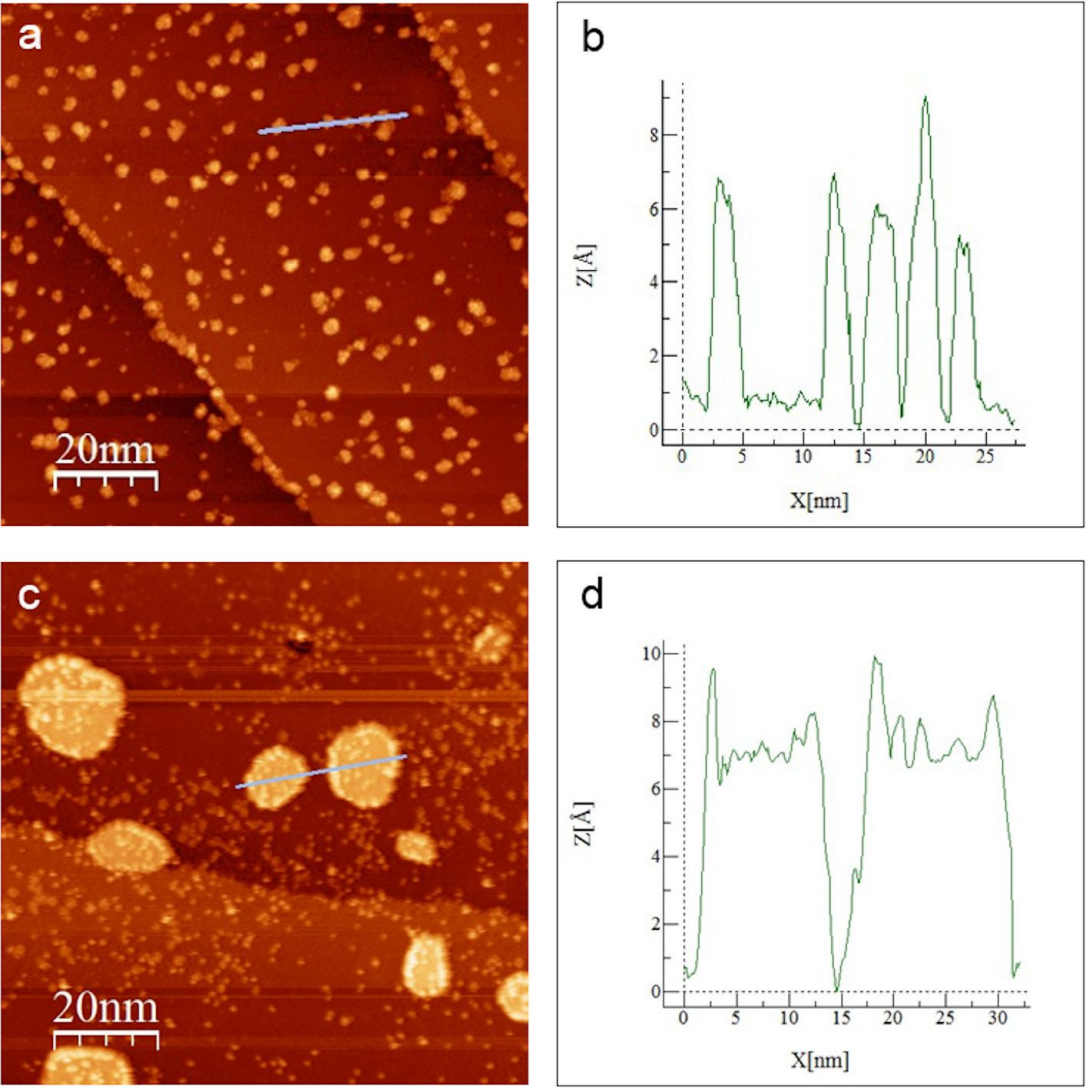
STM images of the initially W_3_O_9_-covered w’-TiO*_x_* phase grown on Pt_3_Ti(111) after annealing the sample for 5 min. (a) Flashing the surface to 600 K leads to cluster agglomerations on the terraces and decoration of the step edges (98 × 98 nm; *U*_B_ = 1.48 V; *I*_T_ = 75 pA). (b) The height profile along the line marked in (a). The cluster agglomerations have a typical thickness between 0.6 and 0.9 nm. (c) Flashing the surface to 900 K leads to the formation of larger WO_3_ islands (98 × 98 nm; *U*_B_ = 1.60 V; *I*_T_ = 75 pA). (d) The height profile along the line marked in (c). The islands have always a thickness of ≈0.6 nm with residues of clusters on top.

Another interesting effect can be observed when the surface covered with W_3_O_9_ is annealed at elevated temperatures. Figure 5a and Figure 5c show the cluster arrangement on the w’-TiO*_x_* phase after flashing the surface for 5 min to 600 and 900 K, respectively. After the annealing at 600 K, hardly any single nanocluster can be found on the surface. In return, the step edges are now highly decorated with clusters, while the terraces are covered by up to 1 nm high and 5 nm wide cluster agglomerations. This indicates a high lateral mobility of the clusters at elevated temperatures, which allows for an energy contact by agglomeration due to strong intermolecular interactions. After annealing the sample at 900 K, the formation of larger islands is observed. The thickness of these islands is always approx. 0.6 nm, which indicates the formation of a WO_3_ double layer. On top of the islands, several small particles, with a maximum height of 0.3 nm, can be found. We believe that these particles are residues of partly decomposed W_3_O_9_ clusters, which suggests that these islands were formed by decomposed W_3_O_9_ agglomerations at temperature values between 600 and 900 K. It should be remarked that, although the height information in STM images is always a convolution of electronic and topographic surface properties, their origin in this case is expected to be strongly dominated by the surface morphology. This is due to the formation of compact oxide island structures with a low influence of molecular electronic features.

Thus, annealing a surface covered with W_3_O_9_ may also be used as a gentle way to grow well-defined WO_3_ islands or thin films. In that context, the observed thickness limitation of two WO_3_ unit cells could be of special interest since the height control in ultrathin film growth is usually challenging by classical deposition methods.

## Conclusion

We showcased the ordered growth of W_3_O_9_ nanoclusters on different ultrathin films of titanium oxide formed by a controlled oxidation of the bimetallic Pt_3_Ti(111) alloy surface. Depending on the characteristics of the oxide phase, either a 1D or a 3D alignment of the clusters was observed by STM. On the rectangular z’-TiO*_x_* phase, the clusters were adsorbed almost exclusively on the numerous holes in the oxide film, forming stripes of single molecules, while on the hexagonal w’-TiO*_x_* phase, the coincidence points between the film and the substrate acted as favored attraction points. In that case, the clusters formed supramolecular assemblies of six hexagonally oriented W_3_O_9_ in the lower layer and one in another layer located on top. The electronic situation of this extraordinary bound cluster enabled an STM tip-induced manipulation, resulting in a partial reduction to a W_3_O_8_ molecule, up to now just theoretically known. At elevated temperatures, the clusters showed a significantly increased lateral mobility, leading first to cluster agglomerations at 600 K and finally to the formation of larger WO_3_ islands by the decomposition of the adsorbed nanoclusters at 900 K. Hereby, the island height was strictly limited to 0.6 nm, which corresponds to a thickness of two WO_3_ unit cells.

Our case study with W_3_O_9_ demonstrates striking possibilities of using the TiO*_x_*/Pt_3_Ti(111) substrate surface for the controlled adsorption and manipulation of metal oxide nanoclusters. Depending on the preparation conditions, different atomically thin and highly ordered titanium oxide films can be formed on the bimetallic alloy surface, offering various templates and bonding options for adsorbates. This approach opens up interesting perspectives for the study of more complex structures of polyoxometalate compounds with multiple stable redox states.

## Experimental

### UHV scanning tunneling microscope

All experiments were performed in an UHV chamber working at a base pressure of 1 × 10^−10^ mbar. Its stainless-steel vessel is equipped with a low-temperature scanning tunneling microscope from Createc, an MCP LEED-Auger electronics from OCI, an IQE 11 sputter gun from Specs, for sample cleaning, and several evaporation sources from Createc. The STM measurements were performed in constant-current mode at liquid nitrogen temperature using chemically etched tungsten tips. The STM images were analyzed with the WSxM software [29].

### Sample preparation

The cleaning procedure of the alloy substrate and the subsequent formation of the two investigated titanium oxide phases were performed in a similar manner as described in [30]. Initially, the Pt_3_Ti(111) single crystal surface (purchased by MaTecK) was cleaned by several cycles of neon sputtering (*p*(Ne) = 1 × 10^−5^ mbar) for 10 min and subsequently annealed at 1200 K for 25 min until a sharp *p*(2×2) pattern was visible by LEED. This procedure led to a clean alloy surface with a single Pt layer termination, which is described in detail in [28]. The z’-TiO*_x_* phase was formed by exposing the clean Pt_3_Ti(111) surface to 150 L of oxygen (150 s at *p*(O_2_) = 1.33 × 10^−6^ mbar), while the sample was kept at a temperature of 1000 K. Exposing the clean Pt_3_Ti(111) surface to an oxygen dose of 600 L (600 s at *p*(O_2_) = 1.33 × 10^−6^ mbar) at a sample temperature of 1000 K led to the formation of the w’-TiO*_x_* phase. The W_3_O_9_ clusters were deposited at room temperature by thermal evaporation of WO_3_ powder with a purity of 99.9% (Sigma-Aldrich) at 840 °C, resulting in a cluster deposition rate of 0.002 monolayer/s. The deposition rate was obtained by previously performed calibration measurements using a quartz microbalance, which was positioned in the same direction and distance toward the evaporation source as the sample in order to obtain comparable results. The surface coverages shown herein represent calculated values based on the used deposition rate. Tests with a varied deposition rate showed no significant impact on the cluster adsorption behavior.

### Density functional theory simulations

The simulations of W_3_O_9_ and W_3_O_8_ clusters were performed using the AMS suite [31]. The geometry optimization and electronic properties were obtained by employing PBE exchange–correlation functional [32] with the D3(BJ) dispersion correction [33–35], the valence triple-zeta polarized (TZP) basis sets composed of Slater-type and numerical orbitals, and scalar zero-order regular approximation (ZORA) [36].
